# Structural MRI Differences in the Thalamus, Caudate Nucleus, and Interthalamic Adhesion in Dogs With Idiopathic Epilepsy

**DOI:** 10.1111/vru.70139

**Published:** 2026-01-26

**Authors:** Tsz Yan Chan, Nicolas Israeliantz, Megan Madden

**Affiliations:** ^1^ Royal (Dick) School of Veterinary Studies University of Edinburgh Edinburgh UK

**Keywords:** basal nuclei, canine, idiopathic epilepsy, subcortical grey matter, thalamus

## Abstract

Structural changes in the thalamus and basal nuclei (BN) have been documented in magnetic resonance imaging (MRI) studies of human epilepsy. Studies that specifically investigate whether equivalent structural changes exist in dogs with idiopathic epilepsy (IE) are limited. This retrospective study evaluated the morphology of the caudate nucleus and thalamus in dogs with IE (*n* = 40) compared to a control group (*n* = 38) using standard MRI studies. Measurements of the interthalamic adhesion (ITA) thickness and area, thalamic area and volume, and caudate nucleus area and volume were made to compare the size and symmetry of these structures between groups. Dogs with IE had a significantly larger ITA thickness (*p* < 0.0001) and area (*p* < 0.0001), smaller thalamic area (*p* < 0.0001) and volume (*p* < 0.0001), and smaller caudate nucleus volume (*p* = 0.0126) than controls. There were no significant differences in caudate nucleus area (*p* = 0.171) and asymmetry index for both thalamus (*p* = 0.104) and caudate nucleus (*p* = 0.853) between groups. Our findings indicate that a smaller thalamic and caudate nucleus size is associated with canine IE. However, further research is necessary to determine whether these findings can be generalized to other BN and to investigate the involvement of thalamocortical or BN‐thalamocortical circuits in the pathophysiology of IE.

**Abbreviations**
AEDs: anti‐epileptic drugs
BN: basal nuclei
Coef.: regression coefficient
ECVN: European College of Veterinary Neurology
fMRI: functional MRI
IE: idiopathic epilepsy
IGE: idiopathic generalized epilepsy
IQR: interquartile range
ITA: interthalamic adhesion
MRI: magnetic resonance imaging
MTLE: mesial temporal lobe epilepsy
NAA: *N*‐acetyl aspartate
ROI: region of interest
T: Tesla
T2‐W: T2‐weighted
VBM: voxel‐based morphometry

## 1 Introduction

Epileptic seizures result from a sudden onset of excessively synchronous neuronal activity, leading to transient motor, autonomic, and behavioral manifestations [1, 2, 3]. Numerous studies have highlighted the role of cortical–subcortical network interactions in seizure genesis and propagation [4, 5, 6]. The subcortical grey matter, comprising the hippocampus, thalamus, and basal nuclei (BN), has a complex anatomical organization [7]. Structural changes in these regions have been well documented in humans with idiopathic generalized epilepsy (IGE), using various magnetic resonance imaging (MRI) acquisition and processing techniques, though findings vary by epilepsy subtype—childhood absence epilepsy, juvenile absence epilepsy, juvenile myoclonic epilepsy, and epilepsy with generalized tonic–clonic seizures—as well as by imaging modality [8, 9]. Beyond IGE, subcortical structural alterations have long been recognized in other forms of epilepsy; such findings are integral to a diagnosis. For example, hippocampal atrophy is a hallmark of mesial temporal lobe epilepsy (MTLE) [10, 11] and correlates with pathological changes observed in resected tissue [12]. In contrast, our understanding of subcortical structural changes in dogs with idiopathic epilepsy (IE) remains limited. Similar to findings in humans with MTLE, MRI studies have shown reduced hippocampal volume [13] and hippocampal asymmetry [14] in dogs with IE. However, research on the thalamus and BN in canine IE is limited. A small study by Huaijantug et al. [15] using standard MRI sequences found no significant differences in thalamic volume between dogs with IE and controls, though the sample size was limited to nine dogs. Meanwhile, a single‐breed pilot study demonstrated grey matter volume reduction in the putamen and claustrum of dogs with IE using voxel‐based morphometry (VBM) [13]. A literature search performed by the authors (PubMed, October 9, 2025), using combinations of the following keywords: “thalamus,” “basal nuclei,” “caudate nucleus,” “subcortical,” “epilepsy,” “canine,” “dog,” and “MRI,” failed to identify additional MRI studies investigating volumetric changes in the thalamus or BN in dogs with IE.

This study aimed to use standard MRI studies to assess morphological differences (area, volume, and symmetry) in the thalamus and BN between dogs with IE and controls. Due to the difficulty in delineating all BN structures on standard MRI sequences, only the caudate nucleus was studied. We hypothesized that the size and symmetry of the thalamus and caudate nucleus would differ between groups. Identifying subcortical structural alterations could enhance our understanding of IE pathophysiology and inform future research on cortical–subcortical network interactions in dogs with epilepsy.

## 2 Methods

### 2.1 Study Design and Case Selection

This was a retrospective, blinded, single‐center study utilizing MRI studies and medical record data collected between 2017 and 2024 at the Hospital for Small Animals, Royal (Dick) School of Veterinary Studies, University of Edinburgh. Ethical approval was granted by the Veterinary Ethical Review Committee, Royal (Dick) School of Veterinary Studies, University of Edinburgh. A power calculation was performed using G*Power (v3.1.9.3, Heinrich‐Heine‐Universität Düsseldorf, Düsseldorf, Germany) to determine the required sample size for IE group and control group at a 0.05 significance level and statistical power of 90% [16]. The anticipated effect size was based on a similar imaging study assessing hippocampal size and symmetry in dogs with IE [14]. This led to a sample size requirement of 37 dogs per group. The institutional database was searched to identify dogs with a Tier II confidence level diagnosis of IE [17]. Brachycephalic breeds were excluded to minimize the influence of skull shape on brain morphology measurements [18, 19, 20]. The control group consisted of dogs presenting to the neurology and neurosurgery service that had neurological examinations consistent with a neurolocalization to the brainstem, cerebellum, or cranial nerves, which had undergone an MRI study of the head. The final diagnoses for dogs in the control group are provided in Table S1. Animals were excluded from the control group if they had a history of epileptic seizures, evidence of forebrain pathology on the MRI or had undergone radiation therapy prior to MRI study. All cases that underwent an MRI study between 2017 and 2024 and met the defined inclusion criteria were included in the study. MRI studies were reviewed, and only patients with complete brain studies were included. All animals in both groups were examined by a European College of Veterinary Neurology (ECVN) specialist or resident‐in‐training. Further information, regarding signalment (age, sex, neuter status, breed, skull morphology [i.e., dolichocephalic versus mesocephalic], and weight), diagnosis, and treatment at the time of referral, were collected from the medical records.

### 2.2 Image Acquisition and Analysis

All MRI studies were performed under general anesthesia with 1.5 T (Siemens Magnetom Avanto 1.5T, Munich, Germany; Philips Intera 1.5T, Amsterdam, the Netherlands; Philips Achieva 1.5T, Amsterdam, the Netherlands). A standard brain study was performed in all cases. The slice thickness was 2.5–3.0 mm, the slice gap was 2.75–3.9 mm, the field of view was 10–30 cm × 10–30 cm, and the matrix size was 192–480 × 192–480. The MRI studies were anonymized, thereby blinding the reviewer to the patients’ group (control versus IE) during data acquisition and statistical analysis. All measurements were performed using Horos software (v3.3.6, Horos Project, New York, USA; https://horosproject.org/) on T2‐weighted (T2‐W) transverse and sagittal images. Six parameters were measured for each patient: interthalamic adhesion (ITA) thickness, ITA area, thalamic area, thalamic volume, caudate nucleus area, and caudate nucleus volume. Forebrain height, area, and volume were also measured for subsequent normalization to reduce the impact of breed variation in brain size. The normalized values of each parameter were presented as a percentage (i.e., regionofinterest(ROI)measurement/forebrainmeasurement×100%). Measurements were performed by the primary researcher, a final year veterinary student (T‐YC), under the supervision of a board‐certified neurologist (MM). The student undertook an initial training period with the supervisor, where the anatomical boundaries of each measurement were demonstrated. The student subsequently performed all measurements. In the initial training period, the supervisor reviewed a random selection of the data to ensure that the regions of interest (ROIs) defined by the student were accurate. When measurements were identified not to be anatomically precise, the supervisor provided feedback, and the measurements were repeated. The supervisor performed a final verification of the measurements on a random selection of data at the end of the data acquisition period.

The aforementioned parameters were manually segmented using Horos software. Different annotation tools within Horos, such as “length” and “pencil,” were used to manually segment the regions of interest. The “ROI manager” provided the measured values. For three‐dimensional measurements, a series of ROIs was manually delineated on consecutive slices using the “pencil” tool and assigned the same label, allowing automated volume calculation via the “ROI volume” tool. This approach was used consistently for all volumetric parameters described below.

**FIGURE 1:**
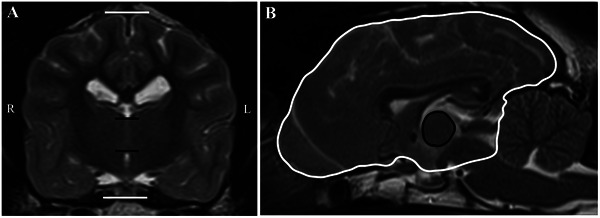
(A) T2‐weighted transverse MRI image at the level of the interthalamic adhesion. Measurement of interthalamic adhesion thickness (between the black lines). Measurement of forebrain height (between the white lines). (B) T2‐weighted mid‐sagittal MRI image. Measurement of interthalamic adhesion area is indicated in black. Measurement of sagittal forebrain area (with parts of midbrain included) is indicated in white using the method described by [23].

The ITA thickness and area were measured using previously described methods [21, 22], where ITA thickness was defined as the linear distance (mm) from the dorsal to the ventral border on the T2‐W transverse image (Figure 1A). The ITA area (mm^2^) was calculated as the area within the boundary defined by the ITA and third ventricle on the T2‐W mid‐sagittal image (Figure 1B). On the same slices, the forebrain height and area were also measured for the subsequent normalization of the ITA thickness and area, respectively. The forebrain height was measured at the level of the midline, from the dorsal boundary of the forebrain to the ventral border of the pituitary gland on transverse view (Figure 1A). The forebrain sagittal area was measured as described by Thames et al. [23], with the boundary between the forebrain and brainstem defined by a line connecting the mammillary bodies and the rostroventral cerebellum in the sagittal view (Figure 1B). This measurement includes a portion of the midbrain in the forebrain sagittal area but allows consistent measurements to be made between patients.

**FIGURE 2:**
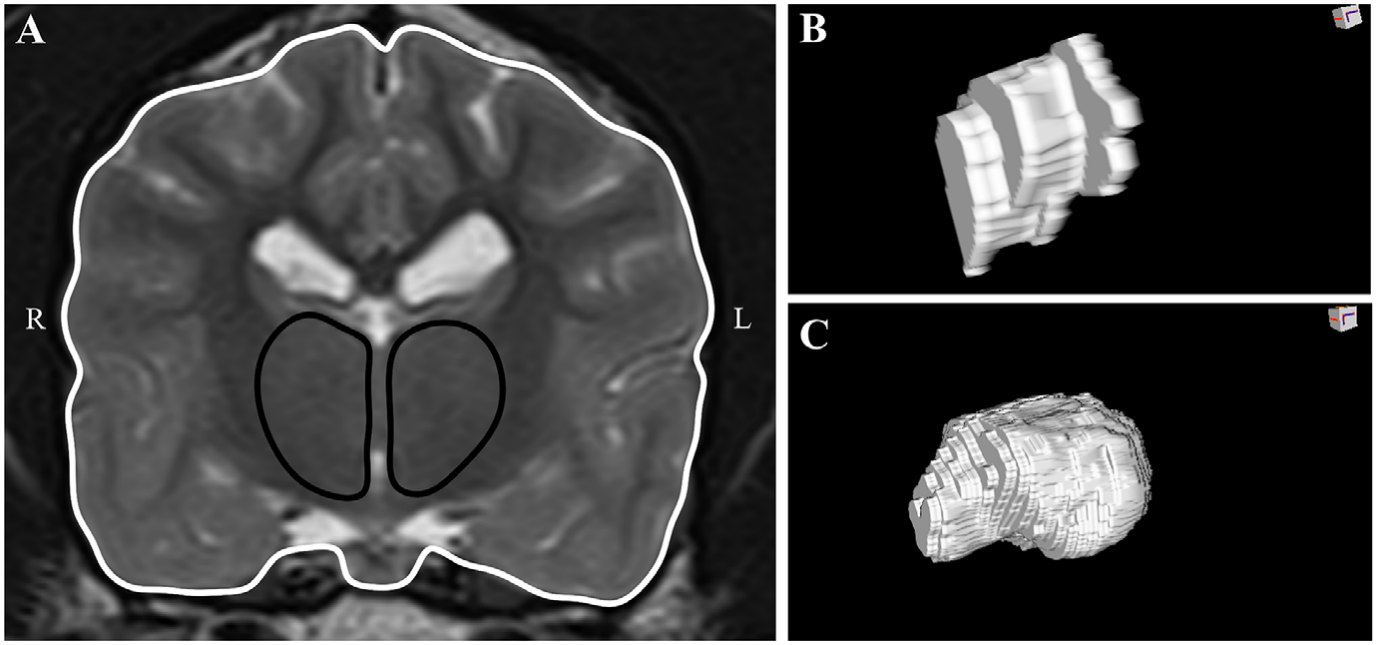
(A) T2‐weighted transverse MRI image at the level of the interthalamic adhesion, showing its thickest point. Measurement of transverse thalamic area is indicated in black. Measurement of transverse forebrain area is indicated in white. (B) 3D volume reconstruction of the left thalamus. (C) 3D volume reconstruction of the forebrain.

The thalamic area (mm^2^) was measured at the level of the thickest point of ITA on transverse T2‐W images (Figure 2A). The ROI was determined by manually tracing the thalamic borders defined by the internal capsule (lateral), lateral ventricles and fornix (dorsal), and hypothalamus (ventral), as described in the previous research [15, 24, 25]. Thalamic volume (mm^3^) was derived from a series of contiguous ROI images spanning the rostral to the caudal boundaries of the thalamus (Figure 2B). Left, right, and total thalamic area and volume measurements were obtained. The corresponding forebrain area (Figure 2A) and total forebrain volume (Figure 2C) were measured on the same transverse T2‐W images for normalization.

**FIGURE 3:**
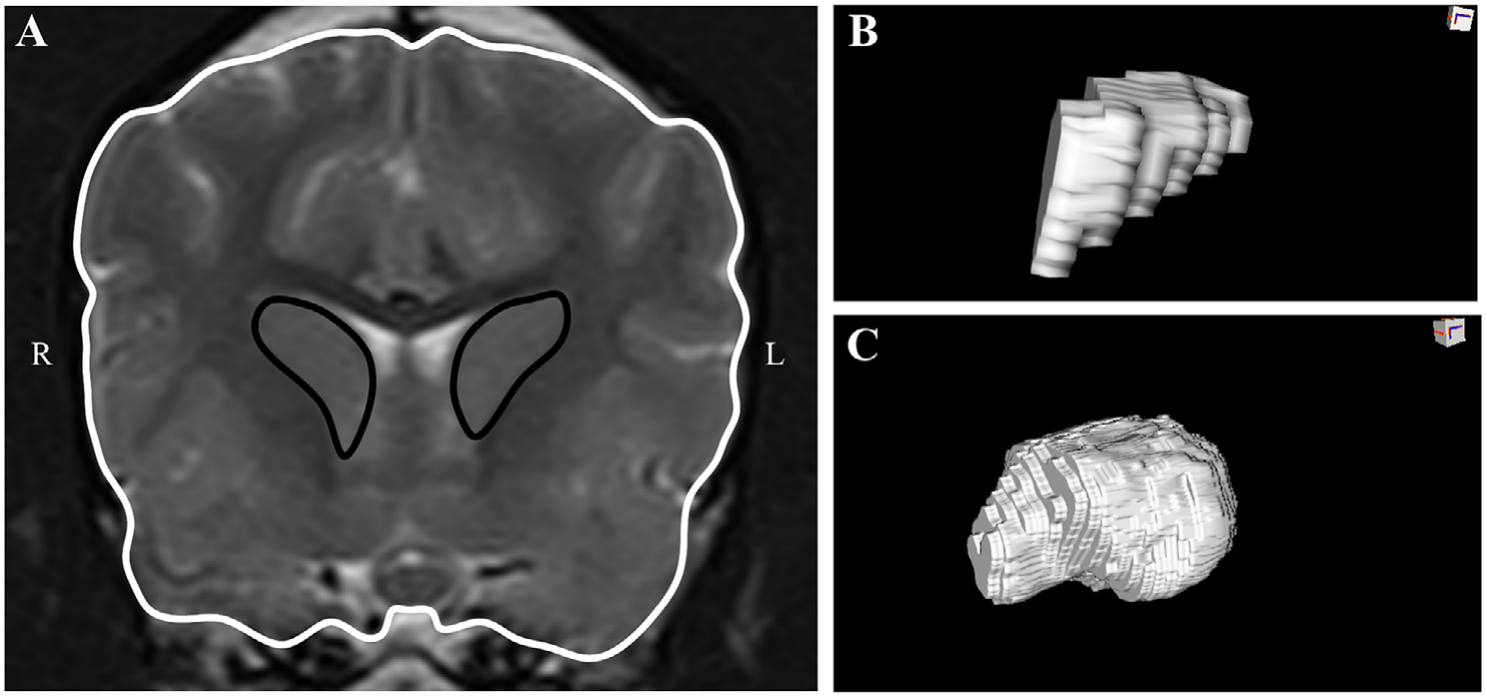
(A) T2‐weighted transverse MRI image at the level of the optic chiasm. Measurement of transverse caudate nucleus area is indicated in black. Measurement of transverse forebrain area is indicated in white. (B) 3D volume reconstruction of the left caudate nucleus. (C) 3D volume reconstruction of the forebrain.

The boundary of the caudate nucleus was defined in accordance with existing MRI atlases [24, 26]. Caudate nucleus area (left, right, and total) was measured at the level of optic chiasm on transverse T2‐W images (Figure 3A). Volumetric assessment was performed on contiguous transverse slices spanning the rostrocaudal extent of the caudate nucleus (Figure 3B). Measurements of the forebrain area and volume were performed in the same manner as described for thalamic normalization. The total caudate nucleus area was normalized to the forebrain area measured at the level of the optic chiasm (Figure 3A), and the total caudate nucleus volume was normalized to the total forebrain volume (Figure 3B,C).

Thalamic and caudate nucleus asymmetry were determined using an asymmetry index previously used to calculate hippocampal asymmetry in epileptic dogs [14]. The index value was calculated using the following equation: absolute difference between the left/right volume divided by the volume of the larger side.

### 2.3 Statistical Analysis

Signalment data (i.e., age, weight, sex, and skull shape) between the two groups were recorded and subsequently compared. Continuous data were normally distributed and, as such, analyzed using Welch's *t*‐test. Categorical data were compared using the Chi‐square test. This approach allows for the identification of potential confounding factors between groups that could introduce bias into the results. The asymmetry indices for the thalamic and caudate nucleus volumes, together with all six normalized parameters (ITA thickness, ITA area, thalamic area, thalamic volume, caudate nucleus area, and caudate nucleus volume) for both groups were assessed for normality using the Shapiro–Wilk test. If the data were normally distributed, the comparisons between IE and control groups were performed using Welch's *t*‐test. If data were not normally distributed, the Mann–Whitney *U*‐test was used. Two‐way ANOVA was performed to evaluate whether factors other than epileptic status (i.e., age, weight, sex, and neuter status) had a statistically significant effect on the measurements. Finally, linear regression was used to investigate the correlation between bodyweight and age with ITA, thalamic, and caudate nucleus size. The null hypothesis stated that there would be no significant differences in the size or symmetry of the thalamus and caudate nucleus between dogs with IE and control dogs. A significance level of 0.05 was applied to all statistical tests to determine whether to reject the null hypothesis. Data were recorded in Microsoft Excel (v16.39, Microsoft, Redmond, WA, USA) and analyzed using Python (Python Software Foundation, Delaware, USA) in Jupyter Notebook (v6.5.4, Project Jupyter, New York, USA) by the main author XXXXX (blinded for review) under the supervision of a board‐certified neurologist (MM). The main author was unblinded to the status of each group (i.e., control or IE) once statistical analysis had been completed.

## 3 Results

### 3.1 Signalment

The age, weight, sex, neuter status, and breed distribution of the two groups are summarized in Table S2. The IE group consisted of 40 dogs, with a mean age of 4.56 years (range: 0.58–10.8 years) and a mean weight of 23.4 kg (range: 3.90–48.0 kg). The group had 3 entire females, 14 neutered females, 6 entire males, and 17 neutered males. This group was composed of dogs from 23 breeds. The mesocephalic breeds included: Cocker Spaniel (4), Springer Spaniel (4), Crossbreed (3), Golden Retriever (2), Husky (2), Hungarian Vizsla (2), Labrador Retriever (2), Australian Kelpie (1), Beagle (1), Cockapoo (1), Flat Coat Retriever (1), Italian Spinone (1), Jack Russell Terrier (1), Nova Scotia Duck Tolling Retriever (1), Miniature Cockapoo (1), Rottweiler (1), Staffordshire Bull Terrier (1), Tibetan Terrier (1), Weimaraner (1), and Yorkshire Terrier (1). The dolichocephalic breeds included: Border Collie (5), German Shepherd Dog (2), and Standard Poodle (1). The control group was composed of 38 dogs, with a mean age of 6.77 years (range: 0.50–12.8 years) and a mean weight of 21.6 kg (range: 2.30–40.2 kg). There were 3 entire females, 12 neutered females, 6 entire males, and 17 neutered males. The group had dogs from 17 breeds. The mesocephalic breeds included: Cocker Spaniel (5), Labrador Retriever (5), Crossbreed (4), West Highland White Terrier (4), Cockapoo (2), Labradoodle (2), Golden Retriever (2), Mongrel (2), Springer Spaniel (2), Beagle (1), American Staffordshire Terrier (1), Flat Coat Retriever (1), Maltese (1), and Patterdale Terrier (1). The dolichocephalic breeds included: Border Collie (3), Greyhound (1), and Lurcher (1). The clinical diagnoses for dogs in the control group are summarized in Table S1. Both groups had a similar distribution of sex and neuter status (*p* = 0.992), skull shape (*p* = 0.612), and body weight (*p* = 0.449). However, the non‐epileptic dogs were significantly older than IE dogs at the time of performing the MRI study (*p* = 0.000820) (Table S2).

### 3.2 Thalamus Measurements

**FIGURE 4:**
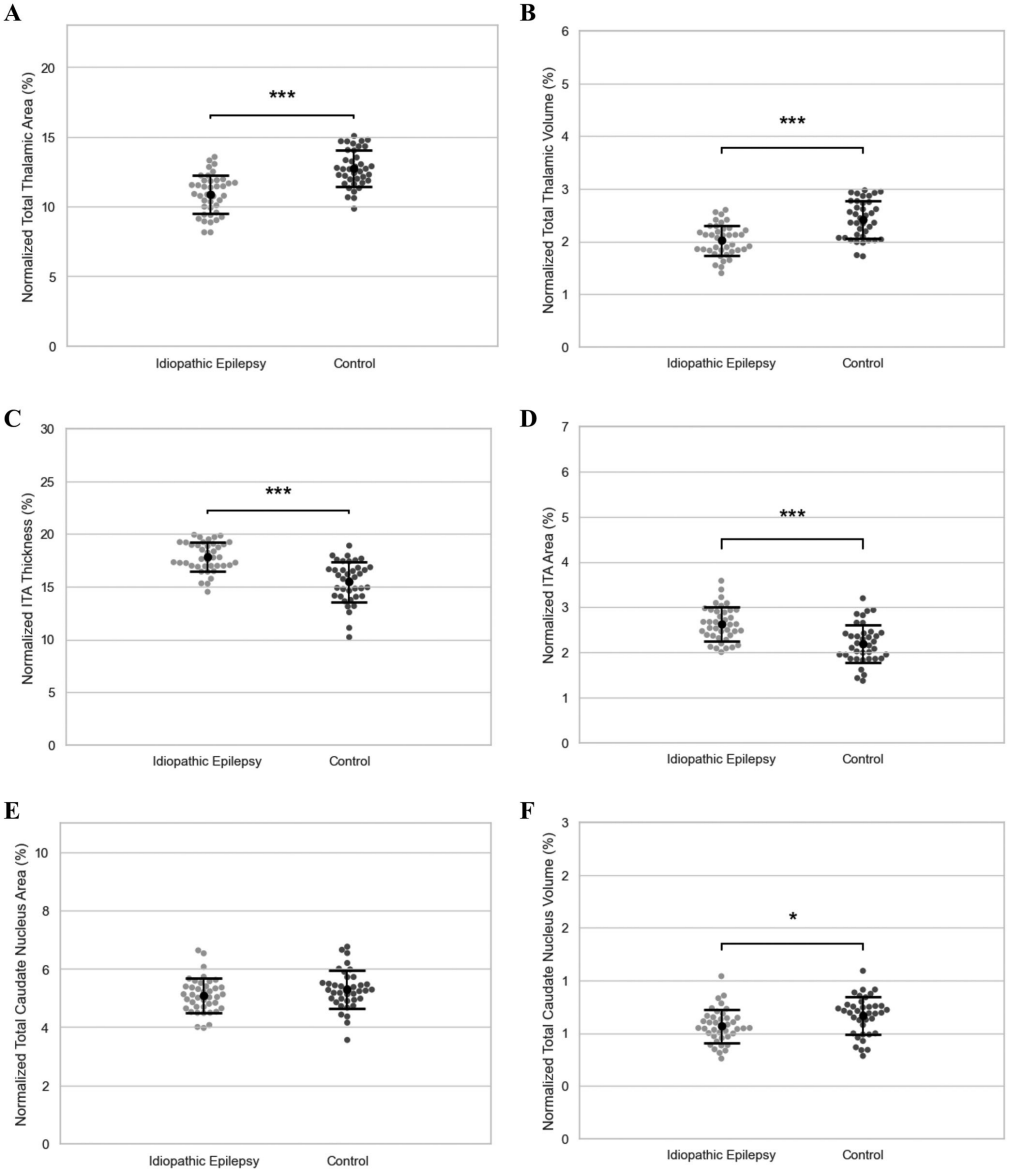
(A) Comparison of normalized thalamic area between groups (10.9% ± 1.38% vs. 12.7% ± 1.30%, *p* < 0.0001). (B) Comparison of normalized thalamic volume between groups (2.02% ± 0.291% vs. 2.42% ± 0.358%, *p* < 0.0001). (C) Comparison of normalized interthalamic adhesion thickness between groups (17.8% ± 1.37% vs. 15.5% ± 1.91%, *p* < 0.0001). (D) Comparison of normalized interthalamic adhesion area between groups (2.63% ± 0.370% vs. 2.19% ± 0.422%, *p* < 0.0001). (E) Comparison of normalized caudate nucleus area between groups (5.09% ± 0.586% vs. 5.28% ± 0.651%, *p* = 0.171). (F) Comparison of normalized caudate nucleus volume between groups (1.07% ± 0.157% vs. 1.17% ± 0.179%, *p* = 0.0126). Sample size (*n*): IE group (*n* = 40), control group (*n* = 38). Error bars represent mean ± standard deviation. Welch's *t*‐test; *p < 0.05, **p < 0.01, ***p < 0.001. IE, idiopathic epilepsy; ITA, interthalamic adhesion.

**FIGURE 5:**
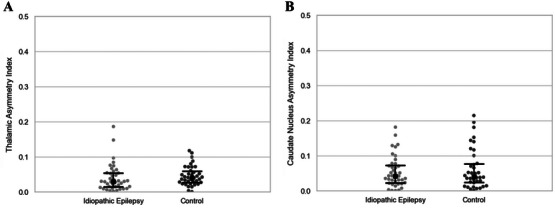
(A) Comparison of thalamic asymmetry index between groups (0.0294 [IQR = 0.0141–0.0539] vs. 0.0406 [IQR = 0.0274–0.0593], *p* = 0.104). (B) Comparison of caudate nucleus asymmetry index between groups (0.0423 [IQR = 0.0228–0.0727] vs. 0.0403 [IQR = 0.0235–0.0771], *p* = 0.853). Sample size (*n*): IE group (*n* = 40), control group (*n* = 38). Error bars represent median and IQR. Mann–Whitney *U*‐test. IE, idiopathic epilepsy; IQR, interquartile range.

**FIGURE 6:**
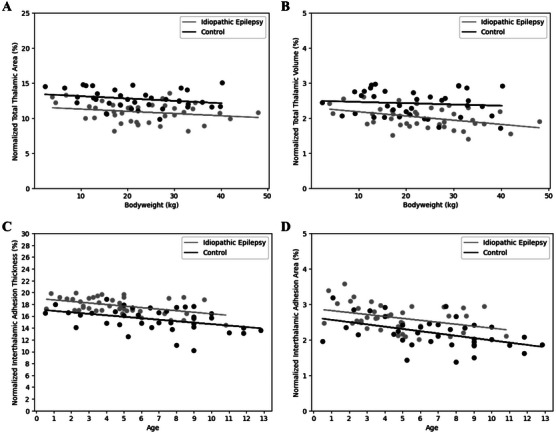
(A) Bodyweight and normalized thalamic area comparison (IE: Coef. = −0.0316, *p* = 0.159; control: Coef. = −0.0336, *p* = 0.129). (B) Bodyweight and normalized thalamic volume comparison (IE: Coef. = −0.0120, *p* = 0.00863; control: Coef. = −0.00360, *p* = 0.559). Sample size (*n*): IE group (*n* = 40), control group (*n* = 38). (C) Age and normalized interthalamic adhesion thickness comparison (IE: Coef. = −0.262, *p* = 0.00245; control: Coef. = −0.249, *p* = 0.0141). (D) Age and normalized interthalamic adhesion area comparison (IE: Coef. = −0.0549, *p* = 0.0224; control: Coef. = −0.0644, *p* = 0.00347). Coef., regression coefficient; IE, idiopathic epilepsy.

Normalized thalamic area (Figure 4A) and volume (Figure 4B) were significantly smaller in the IE group (Table 1). There was no significant difference in the thalamic asymmetry index between groups (Figure 5A and Table 2). Two‐way ANOVA revealed that the values of normalized thalamic area (bodyweight: *p* = 0.0418; IE status: *p* < 0.0001) and volume (bodyweight: *p* = 0.0169; IE status: *p* < 0.0001) were influenced by bodyweight and epileptic status, whereas age, sex, and neutering status had no significant effect on these two thalamic parameters (*p* > 0.05). Subsequent linear regression demonstrated a negative correlation between bodyweight and normalized thalamic area (Figure 6A) with IE dogs having a smaller thalamic area across all bodyweights compared to the control group. A similar trend was observed between normalized thalamic volume and body weight (Figure 6B).

**TABLE 1:** Comparisons of measured thalamic, interthalamic adhesion, and caudate nucleus parameters between idiopathic epilepsy group and control group.

	Idiopathic epilepsy (*n* = 40)	Control (*n* = 38)	*p* value
**Thalamus**			
Normalized thalamic area (%)	10.9 (±1.38)^a^	12.7 (±1.30)^a^	**<0.0001** ^b^
Thalamic area (mm^2^)	191 (±30.2)^a^	228 (±27.5)^a^	**<0.0001** ^b^
Forebrain area (mm^2^)	1760 (±185)^a^	1790 (±182)^a^	0.406^b^
Normalized thalamic volume (%)	2.02 (±0.291)^a^	2.42 (±0.358)^a^	**<0.0001** ^b^
Thalamic volume (mm^3^)	1560 (±301)^a^	1860(±401)^a^	**0.000660** ^b^
Forebrain volume (mm^3^)^c^	78,300 (±14,100)^a^	77,000 (±13,200)^a^	0.694^b^
**Interthalamic adhesion (**ITA)			
Normalized ITA thickness (%)	17.8 (±1.37)^a^	15.5 (±1.91)^a^	**<0.0001** ^b^
ITA thickness (mm)	7.58 (±0.670)^a^	6.58 (±0.845)^a^	**<0.0001** ^b^
Forebrain height (mm)	42.6 (±2.85)^a^	42.6 (±2.79)^a^	0.937^b^
Normalized ITA area (%)	2.63 (±0.370)^a^	2.19 (±0.422)^a^	**<0.0001** ^b^
ITA area (mm^2^)	54.8 (±9.34)^a^	45.2 (±9.47)^a^	**<0.0001** ^b^
Forebrain sagittal area (mm^2^)	2100 (±296)^a^	2080 (±272)^a^	0.769^b^
**Caudate nucleus**			
Normalized caudate nucleus area (%)	5.09 (±0.586)^a^	5.28 (±0.651)^a^	0.171^b^
Caudate nucleus area (mm^2^)	75.2 (±10.8)^a^	75.7 (±8.18)^a^	0.830^b^
Forebrain area (mm^2^)	1480 (±158)^a^	1450 (±179)^a^	0.368^b^
Normalized caudate nucleus volume (%)	1.07 (±0.157)^a^	1.17 (±0.179)^a^	**0.0126** ^b^
Caudate nucleus volume (mm^3^)	827 (±157)^a^	888 (±153)^a^	0.0906^b^
Forebrain volume (mm^3^)^c^	78,300 (±14,100)^a^	77,000 (±13,200)^a^	0.694^b^

^a^ Mean (±standard deviation).

^b^ Welch's *t*‐test *p* value.

^c^ The same volume was used for normalizing thalamic volume and caudate nucleus volume.

Bold values are statistically significant.

**TABLE 2:** Asymmetry index for measured thalamic and caudate nucleus parameters in idiopathic epilepsy and control groups.

	Idiopathic epilepsy (*n* = 40)	Control (*n* = 38)	*p* value
Thalamic volume asymmetry index^a^	0.0294 (0.0141–0.0539)^b^	0.0406 (0.0274–0.0593)^b^	0.104^c^
Caudate nucleus volume asymmetry index^a^	0.0423 (0.0228–0.0727)^b^	0.0403 (0.0235–0.0771)^b^	0.853^c^

^a^ Asymmetry index = absolute difference between the left/right volume divided by the volume of the larger side.

^b^ Median (interquartile range).

^c^ Mann–Whitney *U*‐test *p* value.

### 3.3 ITA Measurements

Compared to controls, the IE group had significantly larger normalized ITA thickness (Figure 4C) and area (Figure 4D) (Table 1). The univariate analysis indicated that both age and epileptic status had a significant effect on the normalized ITA thickness (age: *p* = 0.000410; IE status: *p* = 0.000139) and area (age: *p* = 0.000994; IE status: *p* = 0.00187). Bodyweight, sex, and neutering status had no significant impact on ITA thickness (*p* > 0.05). Linear regression illustrated a negative correlation between age and normalized ITA thickness (Figure 6C) and area (Figure 6D), with the IE group having overall higher normalized ITA values than the control group across age groups.

### 3.4 Caudate Nucleus Measurements

Although there were no significant differences for normalized caudate nucleus area (Figure 4E) and caudate nucleus asymmetry index (Figure 5B and Table 2) between groups, the IE group had a significantly smaller normalized caudate nucleus volume than controls (Figure 4F) (Table 1). Univariate analysis indicated epileptic status as the only factor that had a significant influence on normalized caudate nucleus volume (*p* = 0.00637) with age, weight, sex, and neutering status having no significant impact (*p* > 0.05).

### 3.5 Anti‐Epileptic Treatment in IE Group

Out of the 40 subjects in the IE group, 65% (*n* = 26) were on anti‐epileptic drugs (AEDs) at the time of image acquisitions. All normalized measurements were compared between epileptic dogs with or without AED treatments. No significant differences were found for all the normalized parameters (*p* > 0.05; Table 3).

**TABLE 3:** Comparisons of normalized thalamic and basal nuclei parameters between idiopathic epileptic dogs receiving anti‐epileptic drugs and not receiving anti‐epileptic drugs at the time of imaging acquisition.

	Dogs using AEDs (*n* = 26)	Dogs not using AEDs (*n* = 14)	*p* value
**Interthalamic adhesion (**ITA)			
Normalized ITA thickness (%)	17.7 (±1.25)^a^	18.0 (±1.64)^a^	0.627^b^
Normalized ITA area (%)	2.66 (±0.356)^a^	2.56 (±0.413)^a^	0.444^b^
**Thalamus**			
Normalized thalamic area (%)	10.7 (±1.43)^a^	11.1 (±1.35)^a^	0.383^b^
Normalized thalamic volume (%)	2.03 (±0.308)^a^	2.01 (±0.277)^a^	0.835^b^
**Caudate nucleus**			
Normalized caudate nucleus area (%)	5.03 (±0.601)^a^	5.20 (±0.59)^a^	0.390^b^
Normalized caudate nucleus volume (%)	1.04 (±0.140)^a^	1.08 (±0.140)^a^	0.336^b^

Abbreviation: AEDs, anti‐epileptic drugs.

^a^ Mean (±standard deviation).

^b^ Welch's *t*‐test *p* value.

## 4 Discussion

This study compared the size and symmetry of the thalamus and caudate nucleus between dogs with IE and controls, finding that thalamic area, thalamic volume, and caudate nucleus volume were significantly smaller in the IE group. Both epileptic status and body weight had a significant impact on the thalamic area and volume. Meanwhile, dogs with IE had a significantly larger ITA thickness and area, influenced by both age and epileptic status. These findings allow us to reject our null hypothesis, supporting a role for structures, specifically the thalamus and caudate nucleus, in the pathophysiology of canine IE.

The thalamus is a key relay structure within the thalamocortical circuits, which has been linked to seizure generation and generalization [27]. Structural changes in the thalamus have also been implicated in humans with IGE. Various thalamic atrophy patterns have been identified in different epilepsy subtypes. For instance, atrophy of the medio‐dorsal thalamus has been observed in childhood absence epilepsy [28, 29], whereas both medio‐dorsal and pulvinar atrophy have been reported in patients with generalized tonic–clonic seizures [30]. Furthermore, bilateral thalamic atrophy in human patients with IGE has been shown to be correlated with increased disease duration [31]. Other MRI‐based studies have identified an association between cerebral cortex and thalamic atrophy, suggesting an abnormal thalamocortical connectivity in IGE patients [32, 33, 34, 35]. A role for the thalamus in canine IE is exemplified by recent MRI studies revealing postictal T2‐W and fluid attenuated inversion recovery hyperintense signal in the thalamus, specifically the pulvinar thalamic nucleus [36, 37]—a structure responsible for seizure generation and propagation by interacting with mesial temporal structures in humans [38]. Imaging studies specifically investigating changes in the size of the thalamus in dogs with IE are sparse. A study by Huaijantug et al. [15] used standard MRI sequences and manual segmentation techniques to perform volumetric comparisons of multiple brain regions in nine dogs with IE and four healthy controls. Although no differences in thalamic volume were found between groups, the small sample size in this study is likely to have precluded significant findings. Using an appropriately powered population of dogs, we found a statistically significant reduction in thalamic volume and area in dogs with IE, mirroring findings in human IGE. However, the underlying basis for the thalamic size differences remains unclear. Evidence suggests metabolic and perfusion abnormalities in the thalamus of dogs with IE, including reduced *N*‐acetyl aspartate (NAA) to creatine ratio on magnetic resonance spectroscopy and thalamic hypoperfusion on perfusion‐weighted imaging and single‐photon emission computed tomography [39, 40, 41]. Such changes have been associated with grey matter loss in other neurological diseases [42]. Earlier histopathological reports in epileptic Beagles also describe astrocytic swelling and ischaemic neuronal changes within the cortex, thalamus, and BN [43]. Further work in larger canine populations is needed to clarify the cellular correlates of the structural differences identified in this study.

Interestingly, we found a statistically significant effect of bodyweight on thalamic size, with increasing bodyweight associated with reduced thalamic area and volume. These findings are consistent with research showing higher body mass index in older human adults is linked to lower grey matter volume, including the thalamus [44]. Body condition score was not specifically evaluated in this study. However, it is likely that dogs with larger weights were larger breed dogs, rather than obese animals, and these findings likely reflect breed‐specific variation in thalamic size relative to the forebrain. Further imaging studies using a larger population of healthy dogs would be necessary to investigate this relationship further.

The BN consists of caudate nucleus, putamen, endopeduncular nucleus, globus pallidus, claustrum, and amygdala [26, 45]. These structures form a complex subcortical system that interacts with the thalamocortical circuits [46, 47]. Disruption of the BN‐mediated tonic inhibition over the thalamus can promote seizure generalization [48]. The involvement of BN–thalamocortical pathways in IGE is further supported by reports of progressive atrophy beginning in the globus pallidus and extending to the caudate nucleus, thalamus, and finally, the cortices [49]. Similarly, voxel‐based analyses have revealed regional BN and thalamic atrophy in IGE patients [8], and MRI volumetric studies in MTLE patients indicate reduced bilateral thalamic and striatal volumes [50]. Limited by the ability to accurately define anatomical landmarks of all BN on standard T2‐W images, only the caudate nucleus size was assessed in this study. We observed a smaller normalized caudate nucleus volume in the IE group, though no significant difference in caudate nucleus area was noted between groups, potentially suggesting a regional rather than a generalized reduction in caudate nucleus size. These findings align with human volumetric imaging studies, which have shown a reduced right caudate nucleus and bilateral putamen volume in patients with IGE [51]. Similar changes have also been observed in patients with MRI‐negative cortical epilepsy, where a reduced left caudate nucleus volume was identified on structural MRI, and decreased grey matter density in the right caudate nucleus was observed using VBM [9]. Previous veterinary studies have also demonstrated reduced BN volumes in dogs with IE using VBM, specifically in the claustrum and putamen, but the caudate nucleus was not evaluated [13]. Interestingly, as for the thalamus, alterations in cerebral blood flow to the caudate nucleus have also been documented [39, 40]. Similar pathophysiological mechanisms related to hypoperfusion may explain the altered size of both structures.

In this study, we identified a larger ITA thickness in dogs with IE compared to controls, with statistically significant effect of both epileptic status and age. A negative correlation between age and ITA thickness has been well established in dogs, with measurements of less than 5 mm consistent with brain atrophy [21, 22, 52]. Considering the control group consisted of older dogs, smaller ITA measurements in this group were anticipated and our measurements were found to be comparable to previous studies [21, 22] (Table S3). Despite the age difference between groups, epileptic status produced a statistically significant effect on ITA thickness following univariate analysis. Histological studies have characterized the ITA as a white matter commissure composed predominantly of axons, oligodendrocytes, and glial bridges [53]. Although activity‐dependent myelination has been demonstrated in an experimental rodent model of generalized epilepsy [54], the relevance of this process to ITA enlargement in dogs with IE is unknown. Further histological studies would be required to detail the cellular composition of the ITA in normal dogs and examine differences in levels of myelination and axonal abundance in dogs with IE.

The main limitation of this study is the age difference between groups, due to the typically young age of dogs with IE. Although the control group was older, the subjects were carefully selected to ensure no visible forebrain pathology on MRI, making them an appropriate comparison group. A univariate analysis was also performed to quantify the effect of age on our measurements, and the results showed that epileptic status remained statistically significant. Future studies should aim to use age‐matched controls where possible—this was not possible with the available dataset but could be achieved with a larger multicenter study. Another limitation is the use of the manual segmentation technique, which carries an inherent risk of observer bias. This approach requires expert anatomical knowledge and can be affected by user fatigue and subjectivity. All measurements were performed following standardized protocols and were subsequently reviewed by a board‐certified neurologist. However, intra‐ or inter‐observer reliability assessments were not performed, which would strengthen confidence in measurement consistency. Although the MRI studies were anonymized to blind the measurer to group allocation, some control group subjects had obvious non‐forebrain pathology visible on their scans. These features may have introduced the potential for reader bias. Additionally, slice thickness may affect measurement accuracy by introducing partial volume effects, potentially blurring the boundaries between brain structures. Standardized imaging protocols and consistent measurement procedures across subjects were used to mitigate this, but some degree of measurement variability cannot be completely excluded. Future studies should consider advanced imaging techniques such as VBM, a fully automated method for grey matter volume comparisons, to validate our findings [55]. Automated volumetry offers higher sensitivity than manual methods, making it a promising tool to identify subtle changes in the subcortical grey matter in dogs with IE [56]; it has recently been used to detect changes in temporal lobe volumes in dogs with IE [57]. Although this study found an association between reduced thalamic and caudate nucleus sizes and IE, it remains unclear whether these changes arise as a consequence of epileptic seizures or reflect neurodevelopmental or breed‐related variations that could predispose dogs to IE. This uncertainty is particularly relevant given prior human studies reporting an association between thalamic atrophy and seizure duration [31]. To address this question, longitudinal studies would be required to monitor morphological changes in these structures over time. Finally, our analysis did not reveal an impact of AEDs on thalamic or caudate nucleus size, although treatments and doses were variable between patients (Table S4). Further stratification by epilepsy phenotypes (focal versus generalized epileptic seizures), duration, and treatment regimens in a larger population of dogs would be beneficial to assess the impact of these factors further.

This study identified significant morphological differences in the thalamus and caudate nuclei of dogs with IE compared to controls, mirroring findings from human epilepsy research. Further investigation into BN–thalamocortical circuit activity is needed to elucidate the potential association between these structural differences and the functional networks involved in IE. Better understanding of these relationships could ultimately enhance diagnostic and therapeutic approaches and further establish dogs with IE as a valuable naturally occurring model for human epilepsy.

## Disclosure

The data contained within this manuscript have been presented at the 10th British Veterinary Neurological Society symposium, March 19, 2025. A reporting checklist was not used.

## Conflicts of Interest

The authors declare no conflicts of interest.

## Supporting information




**Supporting Table 1**: Clinical diagnosis of dogs in control group.
**Supporting Table 2**: Age, BW, sex, and breed characteristics of sampled dogs.
**Supporting Table 3**: Comparison of interthalamic adhesion measurements with existing research finding on CT and MRI.
**Supporting Table 4**: Summary of anti‐epileptic drugs (AEDs) treatments and patient distribution.

## Data Availability

The data that support the findings of this study are available from the corresponding author upon reasonable request.
