# Supplementary material for: Structural MRI Differences in the Thalamus, Caudate Nucleus, and Interthalamic Adhesion in Dogs With Idiopathic Epilepsy
Source: Vet Radiol Ultrasound. 2026 Jan 26;67(1):e70139. doi: 10.1111/vru.70139 (PMC12835456; doi:10.1111/vru.70139)
Supplement: Supplementary file 1 — Supporting Table 1: Clinical diagnosis of dogs in control group. Supporting Table 2: Age, BW, sex, and breed characteristics of sampled dogs. Supporting Table 3: Comparison of interthalamic adhesion measurements with existing research finding on CT and MRI. Supporting Table 4: Summary of anti‐epileptic drugs (AEDs) treatments and patient distribution. [file VRU-67-0-s001.docx]

**SUPPLEMENTARY MATERIALS**

| **Diagnosis** | **Number of Patients** |
| --- | --- |
| Polycranial neuropathy | 5 |
| White dog shaker syndrome (idiopathic cerebellitis) | 5 |
| Facial neuropathy | 4 |
| Facial and vestibular neuropathy | 4 |
| Brainstem meningioma | 2 |
| Idiopathic trigeminal neuropathy | 2 |
| Idiopathic vestibular syndrome | 2 |
| Bilateral otitis externa, media and left otitis interna with associated meningitis and multifocal neuritis | 1 |
| Brainstem and cerebellar extra-axial mass | 1 |
| Bilateral lesion affecting tongue innervation | 1 |
| Cerebellar ischemic infarction | 1 |
| Cerebellum neoplasia | 1 |
| Constrictive myelopathy with head tilt on presentation | 1 |
| Hypoglossal nerve sheath tumour | 1 |
| Intoxication presented with blindness | 1 |
| Middle ear cholesteatoma | 1 |
| Meningoencephalomyelitis of unknown origin affecting the brainstem | 1 |
| Retrobulbar neoplasia | 1 |
| Sudden acquired retinal degeneration syndrome (SARDS) | 1 |
| Trigeminal neuritis | 1 |
| Trigeminal nerve sheath tumour | 1 |

Supplementary Table 1: Clinical diagnosis of dogs in control group.

|  | **Idiopathic Epilepsy (n=40)** | **Control (n=38)** | **p-value** |
| --- | --- | --- | --- |
| **Mean Age (year)**  **[min–max]** | 4.56 [0.580 - 10.8] | 6.77 [0.500 - 12.8] | **0.000820^a^** |
| **Mean BW (kg)**  **[min–max]** | 23.4 [3.90 - 48.0] | 21.6 [2.30 - 40.2] | 0.449^a^ |
| **Sex**  **(number of dogs)** | Female entire (3)  Female neutered (14)  Male entire (6)  Male neutered (17) | Female entire (3)  Female neutered (12)  Male entire (6)  Male neutered (17) | 0.992^b^ |
| **Breed (number of dogs)** | Border Collie (5)  Cocker Spaniel (4)  Springer Spaniel (4)  Crossbreed (3)  Golden Retriever (2)  German Shepherd Dog (2)  Husky (2)  Hungarian Vizsla (2)  Labrador Retriever (2)  Australian Kelpie (1)  Beagle (1)  Cockapoo (1)  Flat Coat Retriever (1)  Italian Spinone (1)  Jack Russel Terrier (1)  Nova Scotia Duck Tolling Retriever (1)  Miniature Cockapoo (1)  Rottweiler (1)  Staffordshire Bull Terrier (1)  Standard Poodle (1)  Tibetan Terrier (1)  Weimaraner (1)  Yorkshire Terrier (1) | Cocker Spaniel (5)  Labrador Retriever (5)  Crossbreed (4)  West Highland White Terrier (4)  Broder Collie (3)  Cockapoo (2)  Labradoodle (2)  Golden Retriever (2)  Mongrel (2)  Springer Spaniel (2)  Beagle (1)  American Staffordshire Terrier (1)  Flat Coat Retriever (1)  Greyhound (1)  Lurcher (1)  Maltese (1)  Patterdale Terrier (1) |  |
| **Skull Shape (number of dogs)** | Mesocephalic: 32 (80.0%)  Dolichocephalic: 8 (20.0%) | Mesocephalic: 33 (86.8%)  Dolichocephalic: 5 (13.2%) | 0.612^b^ |

Supplementary Table 2: Age, BW, sex, and breed characteristics of sampled dogs.

^a^Welch's t-test p-value.

^b^Chi-square test p-value.

|  | **ITA Thickness (%)** | **ITA Thickness (mm)** | **ITA Area (mm²)** |
| --- | --- | --- | --- |
| **Current study** | | | |
| Idiopathic Epilepsy (n = 40) | 17.8 (±1.37)^a^ | 7.58 (±0.670)^a^ | 54.8 (±9.34)^a^ |
| Control (n = 38) | 15.5 (±1.91)^a^ | 6.58 (±0.845)^a^ | 45.2 (±9.47)^a^ |
| **Noh et al., 2017** | | | |
| Young | 18.2 (17.4–20.2)^b^ | 6.94 (6.26–7.47)^b^ |  |
| Aging | 17.1 (15.5–19.0)^b^ | 6.27 (5.54–7.27)^b^ |  |
| Cognitive dysfunction | 10.2 (9.8–11.9)^b^ | 3.98 (3.62–4.30)^b^ |  |
| **Hasegawa et al., 2005** | | | |
| Normal (All) |  | 6.79 (±0.70)^a^ | 54 (±11)^a^ |
| Normal (Brachycephalic) |  | 6.37 (±0.52)^a^ |  |
| Normal (Mesocephalic) |  | 7.09 (±0.68)^a^ |  |
| Normal (Dolichocephalic) |  | 7.01 (±0.78)^a^ |  |
| Cognitive dysfunction |  | 3.82 (±0.79)^a^ | 26 (±9)^a^ |

Supplementary Table 3: Comparison of interthalamic adhesion measurements with existing research finding on CT and MRI.

^a^Mean (± standard deviation (SD)).

^b^Median (interquartile range (IQR)).

| **Anti-epileptic Drug (AED) Treatments** | **Number of Patients** |
| --- | --- |
| Phenobarbital | 9 |
| Phenobarbital, Levetiracetam | 5 |
| Imepitoin | 4 |
| Levetiracetam | 3 |
| Phenobarbital, Imepitoin | 3 |
| Phenobarbital, Potassium bromide | 1 |
| Imepitoin, Levetiracetam | 1 |
| **Patients with AED treatments** | **26** |
| **Patients without AED treatments** | **14** |
| Total | 40 |

Supplementary Table 4: Summary of anti-epileptic drugs (AEDs) treatments and patient distribution.
